# Experiences of non-prescribed opioid use during opioid agonist therapy: an interpretative phenomenological analysis

**DOI:** 10.1186/s13722-026-00658-2

**Published:** 2026-07-22

**Authors:** Sarah Clarke, Kelly Holloway, Daniel Werb, Nav Persaud

**Affiliations:** 1 Institute of Health Policy, Management and Evaluation, University of Toronto, Health Sciences Building, 155 College St., Suite 425, Toronto, ON M5T 3M6 Canada; 2 Centre on Drug Policy Evaluation, Li Ka Shing Knowledge Institute, St. Michael’s Hospital, Unity Health Toronto, 209 Victoria St., Toronto, ON M5B 1T8 Canada; 3 Division of Infectious Diseases & Global Public Health, Department of Medicine, University of California San Diego, 9500 Gilman Drive, Mail Code 0507, La Jolla, CA 92093-0507 USA; 4 Department of Family and Community Medicine, St. Michael’s Hospital, Unity Health Toronto, 80 Bond St, Toronto, ON M5B 1X2 Canada; 5 MAP Centre for Urban Health Solutions, Li Ka Shing Knowledge Institute, St. Michael’s Hospital, Unity Health Toronto, 209 Victoria St, Toronto, ON M5B 1T8 Canada

**Keywords:** Opioids, Qualitative, OAT, Opioid agonist therapy

## Abstract

**Background:**

Although the goal of opioid agonist therapy is typically understood by clinicians to be the cessation of the use of non-prescribed opioids, many people taking opioid agonist therapy continue to use other opioids. The purpose of this study was to explore how people who use both prescribed opioid agonist therapy and non-prescribed opioids understand their use of both agents.

**Methods:**

An interpretative phenomenological analysis was conducted with ten participants recruited from harm reduction sites in Toronto who were concurrently using opioid agonist therapy and non-prescribed opioids. Semi-structured interviews were audio-recorded and transcribed verbatim. Data were analysed to explore participants’ understanding of their use of both opioid agonist therapy and non-prescribed opioids.

**Results:**

Participants had unique reasons for the use of opioid agonist therapy and non-prescribed opioids; while opioid agonist therapy is free, relatively safe, and helps control opioid withdrawal symptoms, non-prescribed opioids satisfy opioid cravings, ease physical and emotional pain, and can be used with more autonomy. The participants’ relationships with different opioids are complicated, and some struggle to reconcile their use with their goals.

**Conclusions:**

Participants shared that opioid agonist therapy and non-prescribed opioids fill complementary roles in their lives, each presenting their own benefits and challenges. Their experiences highlight opportunities for improving the provision of opioid agonist therapy in order to make it more effective and acceptable for people who use non-prescribed opioids, which is a crucial strategy in combatting mortality from the opioid toxicity crisis. Opioid agonist therapy can thus be situated within the harm reduction continuum as a practice that can be beneficial whether or not it results in abstinence, and clinicians should offer tailored regimens that take patient preferences and goals into account.

## Background

The opioid crisis in Canada and the United States, marked by increased rates of opioid-related morbidity and mortality that was triggered by illegal marketing of long-acting opioid products, is currently fueled by unregulated opioids [1–4]. Fentanyl has become increasingly prevalent as a direct contributor to opioid-related deaths [5]; it has been the most commonly detected opioid at death in Ontario, Canada since 2014 [4], and its prevalence in cases of opioid-related mortality has increased, rising from 68.1% in 2018 to 89.1% in 2022 [1]. Ontario’s unregulated supply has also become increasingly contaminated with benzodiazepines and other adulterants [6, 7], significantly increasing the risk of adverse outcomes.

One tool to combat opioid-related morbidity and mortality is opioid agonist therapy (OAT), a form of treatment in which a long-acting opioid agonist, usually methadone or buprenorphine, is prescribed. OAT works on a principle of *substitution*: a substance upon which a person has become dependent is replaced with a pharmacologically similar substance that has fewer harmful effects [8]. The mechanism of OAT is to eliminate a person’s need to use non-prescribed opioids through the substitution of a prescribed opioid, thus reducing the risk of harmful consequences. A systematic review and meta-analysis has shown that being on OAT reduces the risk of both all-cause and overdose mortality in people with opioid use disorder (OUD) [9].

Although the use of OAT is intended to replace the use of non-prescribed opioids, many people on OAT have exposures to other opioids [10–12]. While non-prescribed opioid use during OAT is often framed as a “failure” by OAT prescribers [13, 14], it is not clear whether people taking OAT share this view. There have been a small number of studies that examine people’s reasons for non-prescribed opioid use during OAT [15–18]; however, there have not been any explorations of people’s motivations for continuing to take OAT while using non-prescribed opioids. Understanding how people think and feel about their use of each type of opioid will provide insight into the phenomenon of non-prescribed opioid use during OAT; these understandings are crucial for building strategies to maximize the benefits of OAT.

The purpose of this study was to determine how people understand their concurrent use of both prescribed OAT and non-prescribed opioids. We define *concurrent use* as daily or near-daily OAT use combined with at least weekly use of a non-prescribed opioid. The findings might provide insight into how people who use OAT understand and experience how it is prescribed, reveal whether OAT practice meets the needs of people taking it, and inform suggestions to make OAT more supportive.

## Methods

### Methodology

This study uses interpretative phenomenological analysis (IPA) [19]. The IPA approach is phenomenological (i.e., it is about the experience of a phenomenon), hermeneutic (i.e., it investigates how participants make sense of their experience), and idiographic (i.e., it considers cases individually before looking across cases). This approach allows for an in-depth analysis of the meaning that each participant assigns to their experiences of different types of opioid use. This assigned meaning provides insights into participants’ views of the role that each opioid plays in their lives and how the roles intersect.

### Data collection

Data for this study were collected through single one-on-one semi-structured interviews. The questions, which served as a guide for conversations rather than a script, were open-ended and focused on uncovering the meaning that participants assign to their use of prescribed and non-prescribed opioids by asking about their experiences of both, the role of each in their lives, their feelings about them, and the relationship between them. The interviews started with more emotionally neutral questions in order to allow time to develop rapport before moving on to more potentially sensitive questions [10]. Criterion sampling was used for recruitment. The eligibility criteria for participation were being at least 18 years old; being able to speak and understand English; currently taking prescribed methadone or buprenorphine for OUD; having been at a stable OAT dose for at least three months; and regularly (i.e., at least weekly) using at least one other opioid that has not been prescribed to them for at least two months. The target sample size for this study was ten to fifteen participants [19–21].

One author (SC) recruited participants from two harm reduction sites located within community health centres in Toronto. Access to the harm reduction services at these sites is open on a drop-in basis to all individuals who use drugs; the harm reduction services offered include supervised drug consumption, drug testing, naloxone training and kit distribution, access to harm reduction supplies including injection kits, inhalation kits, and safer sex supplies, and education and support delivered by harm reduction staff. After granting permission for recruitment, the harm reduction staff at both sites posted recruitment flyers onsite with the weekly hours during which the author would be present at the site and inviting potential participants to get in touch by phone if they wanted more information. Some participants phoned to confirm a meeting time; others expressed interest in participating while the author was onsite. The author introduced herself to each interested individual and briefly explained the purpose of the study. The author asked all interested individuals about each eligibility criterion and invited those that reported meeting all criteria to participate. Interviews with each participant were conducted in a private office at the recruitment site immediately after consent was obtained and documented; each interview lasted between 17 and 58 min. Participants received $50 in cash following the interview. Interviews were recorded and transcribed verbatim.

### Data analysis

The data were analysed by one author (SC) using the interpretative phenomenological analysis (IPA) approach [21, 22], which seeks to uncover the experience of each participant as well as the breadth of experience across participants. The author read the transcript of the first interview and noted statements that revealed how the participant thought about their use of OAT and non-prescribed opioids. A list of emergent themes was made, then the themes were clustered and relationships drawn between them. This resulted in a table of major themes and sub-themes, each illustrated with key quotes. This process was repeated for each interview transcript. Once all the transcripts were interpreted, a final structured list of major themes representing the whole dataset was created, showing how the participants’ experiences converge and diverge. Each participant was assigned a random pseudonym, and they are referred to using these pseudonyms in order to connect the thread of their personal lived experiences throughout the analysis [22].

## Results

Ten individuals were recruited for this study. We introduce them here based on the information they decided to share with us.

For six of the participants, their first opioids were pharmaceutical:

- Carol, a woman in her fifties living in an apartment, started using opioids over 30 years ago when she was prescribed painkillers following a car accident as a young adult. This led to opioid dependence, which eventually led to her using heroin. She started attending a methadone clinic, which enabled her to stop her heroin use, but a sudden treatment disruption prompted her to return to unregulated opioid use. By this time, the opioid supply had come to be dominated by fentanyl, and upon re-engaging with OAT, she found that methadone was no longer sufficient to control her opioid withdrawal and cravings. Her current prescriber has added slow-release oral morphine to her regimen, which she has found to help her withdrawal symptoms, though she still uses fentanyl occasionally.
- Ruth, an unhoused woman in her forties, became dependent on prescribed opioids by her family doctor for her menstrual cramps as a teenager, and her use continued to escalate even after her family doctor started her on methadone. She found that participation in Narcotics Anonymous provided the support that she needed to stop all opioid use, including methadone; however, her opioid use started again following some destabilization in her life. She has since started on a program of methadone, slow-release oral morphine, and hydromorphone while continuing to use fentanyl occasionally.
- Suzette, another unhoused woman in her forties, was prescribed opioids about 25 years ago for a childhood injury. She was eventually put on buprenorphine, which enabled her to stop, but emotional traumas in adulthood prompted her to start using unregulated fentanyl. She re-engaged with OAT, this time with methadone, primarily as a way to ensure daily access to opioids in case she was unable to obtain fentanyl through sex work and theft.
- Laura, a woman in her thirties living in an encampment, started using her mother’s morphine as a teenager. Her first experience of OAT was following a year of abstinence during incarceration; she was started on methadone shortly after her release. She was weaned off at her doctor’s recommendation, and this led to a return to opioid use. At the time of our interview, Laura had re-engaged with methadone with a new prescriber and was also continuing to use unregulated fentanyl.
- Joyce, an older woman living with her boyfriend, started taking non-prescribed hydromorphone in middle age to manage pain. When she found her use becoming problematic, she started OAT with sublingual buprenorphine. Her OAT clinic had limited weekly hours, and Joyce continued to use non-prescribed hydromorphone to manage her opioid withdrawal when she was not able to make it to the clinic to get her sublingual buprenorphine prescription.
- Maureen, an older woman living in an apartment, developed opioid dependence from non-prescribed hydromorphone use. She was started on the buprenorphine injection while incarcerated, and she found that it completely eliminated her opioid cravings and withdrawal symptoms. However, after she was released from jail, she was not able to get a follow-up injection, and she returned to non-prescribed hydromorphone use. Since then, she had been taking sublingual buprenorphine with the goal of getting back on the buprenorphine injection, which her physician agreed to give her once she stopped her non-prescribed hydromorphone use.

Four of the participants were initiated to opioid use with non-pharmaceutical opioids, either heroin or fentanyl:

- Peter, a man in his sixties living in community housing, started using opioids later in his life. He had been through multiple cycles of fentanyl use, engagement with OAT, discontinuation of OAT, abstinence, and re-engagement with fentanyl.
- Angelina, an unhoused older woman, started using heroin to try to get relief from lifelong anxiety stemming from severe childhood abuse. Since she used theft to be able to afford drugs, she became worried that she would be arrested and would go through opioid withdrawal while incarcerated. She decided to start taking methadone so that she could maintain her access to opioids if she ever went to jail.
- Siddiq, a man in his twenties living temporarily with family after getting kicked out of his partner’s home, started using fentanyl at a young age. He was started on injection buprenorphine, but his fentanyl use escalated significantly after his partner kicked him out, and he was transitioned to methadone. At the time of our interview, his fentanyl use had not decreased.
- Magnus, an older man living in an apartment, had a traumatic childhood that led to early initiation onto heroin. He started on methadone 28 years ago; he was on a very high dose of methadone for almost ten years, and he abstained from other opioids for the entire time, but his methadone was abruptly discontinued due to incarceration. After release, he maintained his abstinence from opioid use until his wife died, when he started taking unregulated fentanyl daily. He also resumed taking methadone, but at a much lower dose.

The overall theme structure for the analysis is presented in Table 1.

**Table 1:** Theme Structure

The purpose of OAT	How well OAT serves its purpose	The purpose of non-prescribed opioid use	The challenges of non-prescribed opioids	How people’s opioid use aligns with their goals
1. OAT can help you stay off other opioids. 2. OAT is insurance against opioid withdrawal.	3. OAT’s effectiveness at controlling withdrawal symptoms and cravings varies. 4. The structure of OAT is difficult to maintain. 5. Methadone is a bad drug.	6. Non-prescribed opioids help you cope with pain and stress. 7. Non-prescribed opioids relieve withdrawal. 8. Non-prescribed opioids relieve cravings.	9. Using fentanyl is dangerous. 10. It is difficult and tiring to make sure you have enough drugs.	11. Some hate opioids. 12. Some want to stop all non-prescribed opioid use. 13. Some are happy with their current opioid use. 14. Some are not sure what to do.

### The purpose of OAT

#### OAT can help you stay off other opioids

Almost all the participants started OAT hoping that it would help them get out of a stressful and escalating cycle of opioid use and withdrawal. Several participants use language evoking this distressing cycle:

I wanted to stop being sick every 2nd, 3rd day. I wanted the merry-go-round to end. (Magnus)

I was tired of pills, tired of pills, pills, buying pills…It was getting a build-up, I was getting higher tolerance…so it was stressful, very stressful for me. (Joyce)

One exception to this pattern is Angelina, whose goal for OAT was to ensure that she would be able to maintain opioid use if she were arrested (see below). However, she knew that she needed to tell her prescriber that she wanted to stop non-prescribed opioid use to gain access to OAT:

I just told [the prescriber] I used heroin and I wanna get off of it. I told him a lie. (Angelina)

#### OAT is insurance against opioid withdrawal

Suzette and Angelina stated that being on OAT was insurance against withdrawal in case they ended up in jail. Both described being afraid of being arrested for stealing; they wanted to be on OAT proactively to ensure ongoing access to opioids and thus avoid withdrawal while incarcerated. Both described getting started on OAT while incarcerated as impossible:

If you go to jail and you’re not on methadone or [buprenorphine], you cannot get it. (Suzette)

The methadone was just there in case I do get caught, then they have to give it to me in jail. Try getting it if you didn’t have it already. You’ll get sick first. (Angelina)

### How well OAT serves its purpose

#### OAT’s effectiveness at controlling withdrawal symptoms and cravings varies

Participants reported varying effectiveness of OAT at controlling opioid withdrawal and cravings. This was likely due to multiple factors, including the non-prescribed opioid they used, the volume of their use, and OAT agent and dose. Some participants experienced complete relief from withdrawal and cravings on OAT. Peter described initially being puzzled by his reaction to sublingual buprenorphine, which he eventually realized was the absence of desire for opioids:

[A]t the very beginning, I remember a feeling, not a high, but a feeling…and I didn’t physically or mentally notice the effects, I just knew I didn’t feel like using fentanyl. (Peter)

Maureen recalled that she had no desire for opioids during a previous experience with injection buprenorphine:

[O]nce I got on [injection buprenorphine], I had no sickness, no cravings, didn’t even think about wanting to do it. (Maureen)

Maureen was subsequently on sublingual buprenorphine, which she found kept her from experiencing withdrawal but did not control her cravings. These cravings drove her to use non-prescribed hydromorphone despite wanting to stop:

Once I get that craving, it’s like, I know, oh fuck. I’m trying to keep my mind off of it because no matter what, I’m not getting over it. Until I have it. (Maureen)

Some participants found OAT to be of limited effectiveness. Suzette saw methadone’s main function as enabling her to get fentanyl:

I know I can get [methadone] every day, it’s already paid for, so in the back of my mind I know, okay, I’m not gonna be really sick, if I can just get that and I can get up, I can go shower, I can put on makeup, I can maybe go find dope. (Suzette)

Suzette found that daily methadone made her well enough to engage in theft and sex work, which helped her afford fentanyl:

Why bother picking up any more methadone? Because I need it, so that I can go out, I can find some kind of hustle. (Suzette)

Suzette thought that methadone’s inadequacy was not unique to her:

I don’t know anyone that’s on methadone that doesn’t still do fentanyl. I don’t know anyone. Every person that I know that goes to my methadone clinic is still on fentanyl…I think that’s the problem, giving us methadone or [buprenorphine]… when there’s all these other drugs in [the supply]. (Suzette)

This mirrored Carol’s experience; while methadone relieved Carol’s withdrawal symptoms when she was using heroin, she found it completely ineffective for fentanyl:

Drinking my methadone, it was like I was drinking water every day. (Carol)

The addition of slow-release oral morphine (SROM) to her methadone made an immediate and significant difference to her withdrawal. Carol echoed Suzette’s statement that methadone is insufficient for people who use fentanyl:

When you’re a fentanyl user, your doctor needs to prescribe the [SROM]…I don’t know why they put them on [methadone] now for fentanyl, because it doesn’t jive with it anymore. (Carol)

#### The structure of OAT is difficult to maintain

Several participants described challenges related to the structures in which OAT is delivered. One well-attested challenge is the burden of witnessed daily dispensing. Siddiq’s description of this evokes the common metaphor of liquid handcuffs [23, 24]:

[Y]ou can’t do nothing on methadone. (Siddiq)

The need to attend the pharmacy daily affected his ability to work (“I can’t get a job and then go to methadone, get a drink…like, it doesn’t work like that”), parent (“You can’t take your son to school”), and do things for himself (“You can’t go on trips”). The structure of methadone therapy was a tether, keeping him from engaging in meaningful activities.

Another challenge of the structure of OAT is the frequency of clinic appointments. Joyce needed to go to her OAT clinic every three days to leave a urine sample and get her prescription, which she found challenging given limited clinic hours:

It’s like…Tuesdays and Thursdays…they’re only there for an hour, two hours, so you gotta be there. (Joyce)

This appointment frequency combined with daily pharmacy visits for witnessed dosing meant that managing OAT took a significant portion of Joyce’s life.

These stories show that the prescriber’s approach plays a significant role in a person’s experience of OAT; a stricter approach to clinic visits and carry doses can create a greater burden, while a more flexible approach can create more room in people’s lives for meaningful experiences. We see the difference in approaches and their impact in Carol’s story. She described her early experiences with methadone as strict and punitive:

[B]ack then…It was just, you’re dirty, you’re kicked off…Like one Christmas, I had [been] clean for two months, or three months, but I had one dirty… It’s a Tylenol 3, it’s not…my choice of heroin…And I was having Christmas…with my family and my kids, and they were denying me carries…I have no problems with Tylenol 3s…but you’re going to punish me to not have Christmas with my family and kids. (Carol)

Carol eventually started seeing a new doctor with a completely different approach to OAT, looking for ways to improve Carol’s life rather than just “enforcing the rules.” The new physician took a non-punitive and innovative approach, including carry doses (despite concurrent fentanyl use) so that she could travel to visit her son:

Instead of any other doctor would be “You’re dirty, you can’t go.” Right? But look at [Doctor], it’s the opposite, total opposite. Six points [of fentanyl] a day, crack all day, and what doctor gives you fucking [OAT] to go on a plane with? No one. But she said, “I know this is going to help you…I know when you’re with your son, there is no drug in your head.” And you know what? When I came back, I was clean, I stayed clean. (Carol)

This incident was completely surprising for Carol, given her previous experience. The prescriber’s flexibility with respect to carry doses had a substantial positive impact; as the prescriber suspected, being able to travel to see her son was deeply meaningful for Carol. This less strict approach significantly changed Carol’s experience of treatment. Carol’s experience appears to be unique among the participants in that no one else described similar examples of flexibility in how their OAT is delivered; within this group of participants, a stricter approach seems to be the norm, with more people describing how OAT hinders their lives rather than how a flexible approach can bring more freedom.

#### Methadone is a bad drug

Most of the participants, both those who had taken it and those who had not, expressed negative opinions about methadone, reflecting a combination of negative experiences with it and the narratives surrounding it. Two participants referred to the belief that methadone was created by Hitler, an obvious condemnation:

Hitler designed [methadone], for God’s sake. (Angelina)

[Hitler] would give it to his soldiers who were injured, so they could go back out and fight without pain, broken limbs, whatnot, and they would still go back out and fight. Broken soldier, as long as you’re alive, you’re a soldier, and that’s what methadone did. (Ruth)

Ruth’s description of methadone use by Nazi soldiers evokes the “zombie” narrative that often surrounds opioid use. Both Angelina and Ruth chose to stay at a low methadone dose (Angelina at 30 mg, Ruth at 45 mg, while a maintenance dose is typically at least 80 mg); this seems to be partly because of ambivalent feelings about methadone’s value and its negative connotations.

Siddiq, who had an extremely negative opinion of methadone (“It ruined my life…It’s just a bad drug”), recounted a story of getting transitioned from injection buprenorphine to methadone, describing the physician’s apparent regret at the switch:

They said “Sorry, we’re unfortunately going to have to put you on methadone.” (Siddiq)

He put emphasis on the word “unfortunately”, suggesting that he thought that the clinician agreed that methadone is objectively a less desirable option than injection buprenorphine.

Participants expressed fewer negative narratives about buprenorphine, perhaps partly because it is newer and therefore has less of a history of stigma surrounding it. Peter, who was offered methadone but was unwilling to try it because of his negative impression of it (“I’ve always for some reason thought ugly of methadone”), described thinking of buprenorphine as a novelty when he first learned about it:

[The nurse practitioner] mentioned [sublingual buprenorphine] and the word “sublingual”, you know, sublingual, it goes under your tongue, and I just liked saying “sublingual.” (Peter)

Similarly, Joyce reported that she was not interested in trying methadone because she had heard too many negative things about it, but she was willing to try buprenorphine because she had heard that it was “better than methadone.” These stories indicate a shared negative perception of methadone as an option for OAT.

### The purpose of non-prescribed opioid use

#### Non-prescribed opioids help you cope with pain and stress

Several of the participants used non-prescribed opioids in response to stressful, painful, or traumatic situations. Suzette had multiple traumas in her past, and her description of her response to fentanyl is compelling:

[Fentanyl] makes everything go away. Every hurt that you’ve ever had in your life, it goes away. It’s like you’re walking on a cloud all the time. You don’t even think about it, you don’t think about people that have died, that you’ve lost, that you loved…I have no pain. No physical pain and no emotional pain. (Suzette)

Peter described fentanyl’s ability to make him feel physically better:

I like the feeling of the relaxation, there’s a comfortableness that I’m not used to…waking up and not hurting. When I wake up, this hurts, that hurts…the pinched nerve, all these things. And they’re not there for a moment… as a pain reliever, as a tool… it works. (Peter)

Carol started using non-prescribed opioids after years of abstinence in response to a traumatic experience with her former methadone prescriber. The period following that disruption in her treatment was characterized by extreme grief and loss, including the death of one of her children and the other child leaving home shortly thereafter, exacerbating her fentanyl use. There was hopelessness in Carol’s description of this period:

When nobody was home to take care of, I didn’t care anymore, so I just dove in… Fuck it. (Carol)

No participants found that OAT provided the same relief. Magnus started using non-prescribed opioids after years of abstinence when his wife died because he needed relief that methadone was not providing:

With the stress that was happening, I would use, when my wife died, I used… I would try methadone to help the cravings, but it doesn’t help cravings. Cravings are mental. (Magnus)

Magnus’s cravings for opioids arose from grief and situations that caused stress. While methadone had always kept Magnus from experiencing withdrawal, it did not give him comfort.

Similarly, Suzette did not find methadone relieved her pain:

The methadone doesn’t take [the pain] away. It just makes it easier for me to go [get fentanyl]. (Suzette)

#### Non-prescribed opioids relieve withdrawal

Withdrawal remained an issue for some participants despite their OAT, and they sometimes used non-prescribed opioids to ease their symptoms at times when they did not have access to OAT. While Laura found that methadone relieved her withdrawal, it did not last a full day; she wanted to start taking a small evening methadone dose but did not feel comfortable requesting it from her doctor. Taking fentanyl either before bed or when she woke up prevented Laura from being in withdrawal when she went to get methadone, making her daily trip to the pharmacy easier:

I know when I use fentanyl before I go to bed, I don’t wake up as sick either. And that is something good. (Laura)

While Joyce found that buprenorphine prevented withdrawal, she often had difficulty accessing her medication. When she was not able to make her clinic visits and get her prescription, she used non-prescribed hydromorphone to manage withdrawal:

Getting there to get the prescription…that was the thing. Sometimes I sleep in, sometimes things happen…It was starting to kick in, the sickness…starting to want to use more of this and more of that…it was not good. (Joyce)

#### Non-prescribed opioids relieve cravings

Maureen and Suzette both used non-prescribed opioids in response to powerful cravings. Maureen repeatedly used the word “need” when discussing her cravings:

By the second, third day… my body is like “Wow, I need it.” (Maureen).

My body craves it so bad that I’m not gonna settle for anything until I get [that hydromorphone or oxycodone], something, I need something. (Maureen)

Suzette described a profound physical need for fentanyl:

I get sick after three hours, I have to smoke it every three hours, I have to. (Suzette)

The intensity of these descriptions shows that cravings can be a powerful motivator for use.

### The challenges of non-prescribed opioids

#### Using fentanyl is dangerous

Several participants had experienced toxicity from unregulated fentanyl:

[Fentanyl]’s a drug that has literally killed me. 40-something times almost. I mean like grey-skin, blue-lip dead. Like I should have been dead. (Peter)

I know I have to not be on fentanyl, I have to. Because someday I will take something too strong and I will die. I’ve overdosed one time, I don’t even remember so I was probably pretty dead. (Suzette)

Despite this perception of fentanyl toxicity as a frightening, dangerous experience, no one mentioned a desire to avoid overdose as a reason for starting OAT. This absence may indicate a resignation to the fact that toxicity is an inevitable risk of using unregulated fentanyl; despite their fear of it, they accept that it may happen. It may also be that people are not aware that OAT reduces overdose risk. If protection against overdose is not a salient goal of people’s OAT use, this represents a significant disconnect between clinicians’ motivations for initiating OAT and people’s motivations for taking it; there may not always be a shared understanding between clinicians and people taking OAT about the purpose of the therapy.

Some participants formerly used heroin, which they reported being difficult to find now. For some, fentanyl was significantly less desirable than heroin:

The only reason I started fentanyl [is] because there is no heroin anymore. (Angelina)

I hate fentanyl but it’s the only thing out there now…All it is that’s the same [between fentanyl and heroin] is it’s opiates…Other than that, it’s totally night and day, the outcome. (Carol)

#### It is difficult and tiring to make sure you have enough drugs

Several participants described challenges accessing drugs. Angelina had to steal to be able to afford fentanyl, which she found morally difficult:

I don’t wanna do crime. I don’t like to steal. I like to earn what I have. And I earn my methadone and my [hydromorphone] because I went to the work to get them. (Angelina)

She preferred her prescribed drugs to fentanyl because her access to them was legitimate; it was preferable to avoid the criminal activities that are a necessary part of acquiring fentanyl.

Joyce described the anxiety of waking up in withdrawal and not knowing where she would get hydromorphone:

I’d wake up, it’s like, how am I gonna get them, where am I gonna get them, am I gonna get them today, ‘cause the sick feeling starts to come, it’s awful, it’s terrible. That was stress, to get the sickness. (Joyce)

Being unsure of where her hydromorphone would come from with the terror of withdrawal hanging over her was a source of significant stress for Joyce.

### How people’s opioid use aligns with their goals

#### Some hate opioids

Angelina hated opioids but was afraid to stop because of withdrawal. She described all the drugs that she was using (methadone, hydromorphone, fentanyl, and crack), as “bad”:

They’re all bad for us…[T]hey’re strange, they’re not the right chemicals to fix myself. (Angelina)

She saw no difference between methadone and other opioids (“To me it’s just like a drug too, that methadone”). She wanted to use as little as possible, taking methadone whenever possible, hydromorphone if she could not get methadone, and fentanyl when she could not get either.

Siddiq also hated all opioids; he described no positive aspects of his use of fentanyl, and he denounced methadone as “a bad drug”. He wanted to stop his use of all opioids, but he knew that he needed OAT to stop using fentanyl, although he profoundly resented the restrictions that it placed on his life:

[Methadone] ruined my life… Going to methadone every day, you can’t do anything else…you can’t do nothing on methadone. (Siddiq)

Siddiq’s goal was to stop his fentanyl use and then wean off methadone as soon as possible, since using opioids, including methadone, was not in line with his goal of “living a normal life”:

[B]eing on all this stuff is not living a normal life. (Siddiq)

This feeling is attested among young people who use opioids; a qualitative study of street-involved youth in Vancouver [25] found that participants generally viewed long-term use of OAT as undesirable and incompatible with having “a normal future”, mirroring Siddiq’s words exactly. Like Siddiq, the young people in this study saw OAT as a short-term intervention that ideally leads to “full recovery” in the form of complete opioid abstinence rather than longer-term management of a chronic condition, as it is generally understood by prescribers.

Joyce also wanted to stop all opioid use. She was confident that being on injection buprenorphine would help her eliminate hydromorphone use, but even before starting it, she was already looking ahead to stopping it. While she had a pragmatic attitude about it (“If I have to, I will continue to take whatever helps me”), she seemed wistful:

I’m just wondering if I’ll ever be okay without it, that’s all. (Joyce)

While being on injection buprenorphine was a price that she was willing to pay to be able to stop using hydromorphone, she would prefer not to be on any opioids.

#### Some want to stop all non-prescribed opioid use

Carol’s goal was to stop using fentanyl and use only methadone and SROM; she still used fentanyl sometimes but less than she used to, and she felt that she was on a good trajectory:

[I’m] getting my shit together for [my son’s] wedding, and I know I will. My goal is to just be doing my prescription drugs. (Carol)

Maureen wanted to be on injection buprenorphine, which she was confident would let her stop hydromorphone:

I’m gonna get through the next week hopefully, to get…[the injection]. ‘Cause I know once I get on that, it’s smooth sailing. (Maureen)

Ruth could not imagine being without opioids, and her ideal scenario included some opioid use:

[My goal is to be] still using, but not completely dependent. (Ruth)

Though she disliked methadone, she sounded satisfied with her safer opioid supply regimen; she found that SROM and hydromorphone helped her cut her fentanyl use significantly:

I take 700 mg [of SROM] at 10 or 11 in the morning, and they last me until I wake up the next morning, and I’m actually not dopesick…The [hydromorphone is] breakthrough…Honest answer? When I wanna get high, that’s what I can take. They give me that little boost. (Ruth)

Ruth would prefer to use only prescribed opioids, and she felt that SROM and hydromorphone were moving her closer to that goal.

#### Some are happy with their current opioid use

Laura was satisfied with her regimen. She described her biggest challenges as difficulties affording fentanyl and living far from her methadone pharmacy, but she seemed happy with the drugs themselves. When the interviewer asked Laura how she felt about her use of methadone and fentanyl, her reply was straightforward:

[I’m] maintaining. I find I’m doing very well. (Laura)

Magnus was also happy with his use. He said that while his extremely low methadone dose (20 mg) helped him cut down, using fentanyl was essential:

I don’t want to stop. I’m at a crucial time in my life, after losing my wife, and I need that support for pain in my mind. (Magnus)

He was happy with his regimen and expressed confidence that he could stop if he chose:

I’m where I’m at, ‘cause that’s where I wanna be at… I’m at where I’m sufficient. I don’t quit drugs ‘cause I don’t want to. If I wanted to, I could. (Magnus)

#### Some are not sure what to do

Peter could not decide how to move forward. Fentanyl use made him feel guilty, but he felt unable to commit to OAT. He repeatedly asserted his desire to stop his opioid use (“I’m at that point again where it’s gotta stop”; “I’ve just gotta stop everything”; “This little tirade has to end somehow”); however, he seemed unconvinced that he was going to follow through:

Well, I mean, the plan [to see the doctor]…that’s been said many many times over the past year. (Peter)

When the interviewer asked Peter what his ideal scenario would be, he hesitated:

I wanna say sobriety, but…I don’t think I’ll ever be completely clean, at this point in my life…I guess I want more time between usages. (Peter)

Suzette was tortured by the situation in which she found herself. She repeatedly referred to herself and her circumstances as “disgusting” and “gross” and was consumed with self-loathing and guilt, but she was terrified at the thought of not using fentanyl:

It’s a disease, it’s such a bad disease, but I don’t want off of it. I’m afraid. (Suzette)

I’m so depressed without my dope, like I need dope. (Suzette)

Suzette’s description of the role of fentanyl in her life made it seem like both the greatest burden she had and the only thing she cared about.

## Discussion

### Key findings

The global themes arising from the data yields the following findings regarding concurrent use of prescribed OAT and non-prescribed opioids: the purposes of OAT and non-prescribed opioids can be complementary; autonomy can be exercised in creative ways; and OAT optimization is important for the participants, although it may still not meet the needs that non-prescribed opioid use meets.

The participants’ reflections on the roles of OAT and non-prescribed opioids in their lives revealed that, for most of them, neither agent alone can provide what they need. The advantages and disadvantages that the participants identified of OAT and non-prescribed opioids are supported by the literature. OAT is advantageous for people because it is free, relatively safe, can be accessed daily, and can help control withdrawal [26, 27]; it can also be insurance against potential future withdrawal [28]. However, there are restrictions on when and where it can be used [23, 29], not everyone finds it effective at controlling withdrawal and cravings, especially people who use fentanyl [18, 30–32], and some have negative views of it, particularly methadone [33, 34]. The participants reflected that while non-prescribed opioids satisfy cravings, ease pain and stress, and can be used with more autonomy, they are expensive, can be dangerous, and can be difficult and time-consuming to acquire [18, 35, 36]. While some participants shared goals of eventually changing their patterns of use, there were factors that made the use of both prescribed OAT and non-prescribed opioids important. These types of use are thus complementary, satisfying different needs.

Patient autonomy has been identified as a crucial factor in OUD treatment [37–41], and there are qualitative studies exploring how people exercise autonomy within the often-strict constraints of OAT [42, 43]. This desire for autonomy is shown in the results; several participants demonstrate autonomy in their treatment by finding creative ways to use OAT to help them achieve their self-identified goals. For example, Angelina, Ruth, and Magnus all chose to remain on low methadone doses. Current recommendations that see the elimination of non-prescribed opioid use as the treatment goal would suggest that their methadone doses should be increased; however, this would not be in line with their goals. Angelina and Suzette both used their methadone prescriptions as insurance against withdrawal in jail. Peter made an opportunistic decision to eliminate all opioid use by discontinuing his buprenorphine rather than restarting it at a lower dose. These participants found creative ways to exercise some control over their care in line with their goals.

The participants’ descriptions of their experiences revealed instances where a different clinical approach to OAT might have been helpful. Joyce and Maureen reported being told that they could not receive the buprenorphine injection until they had used only sublingual buprenorphine for least eight days. However, although there is a requirement that people must have at least seven days of buprenorphine use before receiving the injection [44], there is nothing in the product monograph that indicates they cannot use other opioids. Maureen felt that the buprenorphine injection would eliminate her need to take hydromorphone, but she would not receive the injection until she stopped taking hydromorphone. Joyce’s challenge was attending her clinic twice a week to obtain her buprenorphine prescription; she used hydromorphone when she missed her appointments. These difficulties would be mitigated if she were given a prescription for a week’s worth of buprenorphine. Laura described needing to take fentanyl to sufficiently ease her withdrawal to get to the pharmacy to receive methadone; this might be mitigated if she had take-home methadone doses. Suzette found that methadone alone did not eliminate her cravings for fentanyl; the addition of SROM or a short-acting opioid like hydromorphone might provide greater relief from fentanyl cravings. The participants receiving OAT that included some of these techniques (Carol and Ruth were taking SROM in addition to methadone, and Angelina and Ruth were being prescribed hydromorphone) described finding them beneficial.

These examples illustrate the importance of optimizing the provision of OAT and tailoring the clinical approach to the individual [45–47] without the expectation that this will necessarily lead to a cessation of use. Guidance for initiating injection buprenorphine, flexible take-home OAT doses, adding SROM to methadone, and combining short- and long-acting opioids to reduce unregulated opioid use can be found in recent recommendation documents [48–53]. Through OAT, these participants are trying to solve a problem in their lives, and they are helped and hindered in various ways by their OAT delivery. Although it is not clear from the participants’ accounts why particular clinical choices have been made, more flexible and person-centered OAT would potentially maximize the benefits of the therapy in a way that aligns with their goals, whether or not these goals include the cessation of non-prescribed opioid use. A tailored approach requires clinicians and people taking OAT to have a shared understanding of the purpose of the therapy. A great deal of research and clinical guidance on substance use has been predicated on the medical or “brain disease” model of addiction, which holds that addiction is a “chronic, relapsing disease of the brain” [54], albeit one that is influenced by social and personal factors. The intention behind this model was to reframe addiction as a health condition rather than a moral failing, thus reducing the associated stigma. However, this framing of OUD does not resonate with everyone; some individuals who seek medical treatment for opioid-related concerns—Siddiq, for example—may hold a different narrative about their opioid use, and OAT may not be desired as a long-term intervention [25]. A tailored approach to OAT requires that the clinician understands the individual’s narrative about their opioid use and their goals.

### Implications

There is an urgent need to address the opioid crisis that is now in its “third wave” [55], characterized by a toxic unregulated opioid supply that continues to claim thousands of lives each year in Ontario. The harms of this toxic supply are exacerbated by the fact that harm reduction–oriented services for people who use drugs are closing [56–59], putting people at increased risk of death. The dismantling of these services, which have been shown to reduce overdose rates and improve outcomes for individuals who use them [60, 61], is leaving people who use opioids with fewer options for safer use.

OAT is protective against opioid-related mortality [9, 62]; thus, it is essential to ensure that it is readily available to people who want it and to maximize retention through patient-centered approaches [63], including adapting titration and dosing to the toxicity of the unregulated opioid supply [31, 51, 64]. The key findings above—the use of different agents to meet different needs, the desire for autonomy in treatment, and the importance of flexible, tailored therapy—can all be applied as strategies to increase the effectiveness and acceptability of OAT to people who use opioids.

In addition to the challenges associated with OAT, the participants described other circumstances, including poverty, loss, and trauma, that are unlikely to be resolved through their treatment. Many of these issues are largely outside the scope of practice of OAT clinicians and require broader structural solutions. However, improvements to OAT practice would likely make a material difference in the lives of these participants.

There is no single strategy that will bring an end to the opioid crisis, and it has been noted that an exclusively medical approach, particularly one that is focused on treatment and recovery, will not reach everyone at risk of adverse effects from the toxic unregulated drug supply [65]. Nonetheless, comprehensive, patient-centered OAT is a crucial tool that prescribers can offer to reduce people’s risk of opioid-related mortality, whether or not it results in the cessation of non-prescribed opioid use. OAT can thus be understood as a part of the harm reduction spectrum; people can derive benefits from it even if they do not intend to pursue abstinence. Prescribers should be supported to provide OAT that considers each individual’s needs, challenges, and goals through flexible care that emphasizes agency and choice.

### Limitations

The IPA approach to inquiry is idiographic; its findings are specific to the participants rather than generalizable. The insights this study provides into the experience of using non-prescribed opioids during OAT are specific to the ten individuals under investigation, all of whom were recruited from two specific harm reduction sites in Toronto. These findings represent a single interpretation of the descriptions of the experiences of these particular individuals as they understood them at the time of the interview and are not representative of all individuals concurrently using prescribed OAT and non-prescribed opioids. This approach does not provide information about the prevalence of experiences or circumstances.

This study does not explore how participants’ experiences might have been influenced by facets of their identities (e.g., race, gender, sexuality). People who use drugs are a highly marginalized group, and this experience of marginalization can be compounded by other intersecting axes of oppression. The current study does speak to any experiences that are unique to a particular sociodemographic group.

## Conclusion

This interpretative phenomenological analysis study contributes to the literature by providing insights into the experience of concurrently using prescribed OAT and non-prescribed opioids through the interpretation of the lived experiences of ten individuals; this study is the first to explicitly consider the unique reasons participants have for both their non-prescribed opioid use and their OAT use. The participants shared that OAT and non-prescribed opioids fill complementary roles in their lives, each presenting their own benefits and challenges; the participants’ relationships with different opioids are complicated, and some struggle to reconcile their use with their goals. Their stories highlighted opportunities for improving the provision of OAT to make it more effective and acceptable for people who use non-prescribed opioids, which is a crucial strategy in combatting mortality from the opioid toxicity crisis; clinicians should offer tailored OAT regimens that take patient preferences and goals into account. Recent recommendation documents have provided guidance for approaches that incorporate these strategies; future research should focus on measuring on the uptake of these recommendations and their impact on the satisfaction, treatment retention, and outcomes of people taking OAT.

## Data Availability

The dataset generated and analysed during the current study are not publicly available due to the terms of the informed consent form signed by the participants.

## Declarations

### Authors’ information

This research was conducted as SC’s Master’s thesis at the Institute for Health Policy, Management and Evaluation at the University of Toronto under NP’s supervision with KH and DW as committee members. MAP Centre for Urban Health Solutions (NP), Department of Family and Community Medicine, University of Toronto (NP), Department of Family and Community Medicine, St. Michael’s Hospital, Unity Health Toronto (NP).

## Abbreviations

IPA: Interpretative phenomenological analysis
OAT: Opioid agonist therapy
OUD: Opioid use disorder
SROM: Slow-release oral morphine
